# Amplification and Over-Expression of the *MDM2* Gene in Human Soft Tissue Tumours

**DOI:** 10.1080/13577149778434

**Published:** 1997-03

**Authors:** Helen Patterson, Diana Barnes, Sandra Gill, James Spicer, Cyril Fisher, Merion Thomas, Robert Grimer, Chris Fletcher, Barry Gusterson, Colin Cooper

**Affiliations:** 1 Section of Molecular Carcinogenesis Institute of Cancer Research Haddow Laboratories 15 Downs Road, Belmont, Sutton Surrey SM2 5NG UK; 2 Imperial Cancer Research Fund Clinical Oncology Unit Guys Hospital London UK; 3 Department of Histopathology Royal Marsden Hospital London UK; 4 Department of Surgery Royal Marsden Hospital London UK; 5 Department of Surgery Royal Orthopaedic Hospital Birmingham UK; 6 Department of Histopathology St Thomas's Hospital London UK; 7 Section of Cell Biology and Experimental Pathology Institute of Cancer Research Surrey UK

## Abstract

*Purpose*. Amplification of genetic sequences on chromosome 12q13 is frequently found in soft tissue tumours. However, for the *MDM2* gene, over-expression of the MDM2 protein has not always been shown to accompany gene amplification, raising the possibility that amplification of genetic sequences targets alternative genes on chromosome 12q13 for over-expression. To investigate this discrepancy, we have examined 129 soft tissue tumours for amplification of the *MDM2* gene using Southern analysis, and 39 of these tumours were also examined by immunohistochemical staining for MDM2 over-expression.

*Results*. Gene amplification was identified in 14/114 (12.3%) of the malignant tumours, but was not identified in any of the benign tumours; 21/39 (54%) of the malignant tumours also demonstrated MDM2 over-expression. Within this group the *MDM2* gene was over-expressed in every tumour in which the gene amplification was found, and over-expression in the absence of gene amplification was also found in an additional 10 tumours.

*Discussion*. These data demonstrate a clear correlation between the presence of *MDM2* amplification and MDM2 over-expression, and provide persuasive evidence therefore that the amplification of genetic sequences on chromosome 12q13 in soft tissue sarcomas targets the *MDM2* gene for over-expression. These data also indicate that alternative mechanisms may contribute to MDM2 over-expression within some tumours.


*Sarcoma* (1997) **1**, 17–22

ORIGINAL ARTICLE

### Amplification and over-expression of the *MDM2* gene in human soft tissue tumours

HELEN PATTERSON,[1] DIANA BARNES,[2] SANDRA GILL,[1] JAMES SPICER,[2] CYRIL FISHER,[3] MERION THOMAS,[4] ROBERT GRIMER,[5] CHRIS FLETCHER,[6] BARRY GUSTERSON[7] & COLIN COOPER[1]

[1]*Section of Molecular Carcinogenesis, and* [7]*Section of Cell Biology and Experimental Pathology, Institute of Cancer Research, Surrey,* [2]*Imperial Cancer Research Fund Clinical Oncology Unit, Guys Hospital, London,* [3]*Department of Histopathology, and* [4]*Department of Surgery, Royal Marsden Hospital, London,* [5]*Department of Surgery, Royal Orthopaedic Hospital, Birmingham &* [6]*Department of Histopathology, St Thomas's Hospital, London, UK*

**Abstract**

*Purpose.* Amplification of genetic sequences on chromosome 12q13 is frequently found in soft tissue tumours. However, for the *MDM2* gene, over-expression of the MDM2 protein has not always been shown to accompany gene amplification, raising the possibility that amplification of genetic sequences targets alternative genes on chromosome 12q13 for over-expression. To investigate this discrepancy, we have examined 129 soft tissue tumours for amplification of the *MDM2* gene using Southern analysis, and 39 of these tumours were also examined by immunohistochemical staining for MDM2 over-expression.

*Results.* Gene amplification was identified in 14/114 (12.3%) of the malignant tumours, but was not identified in any of the benign tumours; 21/39 (54%) of the malignant tumours also demonstrated MDM2 over-expression. Within this group the *MDM2* gene was over-expressed in every tumour in which the gene amplification was found, and over-expression in the absence of gene amplification was also found in an additional 10 tumours.

*Discussion.* These data demonstrate a clear correlation between the presence of *MDM2* amplification and MDM2 over-expression, and provide persuasive evidence therefore that the amplification of genetic sequences on chromosome 12q13 in soft tissue sarcomas targets the *MDM2* gene for over-expression. These data also indicate that alternative mechanisms may contribute to MDM2 over-expression within some tumours.

**Key words:** MDM2 *gene, amplification, over-expression, soft tissue sarcoma.*

### Introduction

A variety of mammalian tumour cells contain amplified copies of genes whose transforming potential is then activated by their concomitant over-expression. In keeping with this, double minute chromosomes and homogeneously staining regions have been identified in malignant fibrous histiocytomas, liposarcomas, and alveolar and embryonal rhabdomyosarcomas.[1–3] A cluster of genes which map to chromosome 12q13 have been found to be amplified and over-expressed in soft tissue sarcomas, and include the *MDM2, GLI, CDK4, GADD153* (CHOP) and *SAS* genes.[4–8]

The *MDM2* gene was originally identified as a dominantly transforming oncogene amplified and over-expressed from double minute chromosomes in the 3T3DM cell line.[9] *MDM2* was subsequently shown to cooperate with *RAS* in the transformation of primary rat embryo fibroblasts, and to be amplified in liposarcomas, malignant fibrous histiocytomas (MFHs) and osteosarcomas.[4] MDM2 has been shown to bind p53 and inhibit p53-mediated transactivation[10] and is itself a target for p53-mediated gene regulation via a *cis*-acting p53 response element in its first intron.[11] Elevated p53 expression results in increased levels of MDM2 mRNA and protein, which in turn binds to and modulates p53 function. It has now also been shown that MDM2 can bind and inhibit the growth regulatory function of RB1[12] and can activate E2F1/DP1 transcription factors which are involved in promoting entry into the S-phase.[13] The oncogenic activity of MDM2 may therefore derive from its ability both

Correspondence to: H. Patterson, Section of Molecular Carcinogenesis, Institute of Cancer Research, Haddow Laboratories, 15 Downs Road, Belmont, Sutton, Surrey SM2 5NG, UK. Fax: +44 181 643 0238.

1357-714X/97/010017-06 ©1997 Journals Oxford Ltd to repress p53 function and to stimulate the activity of S-phase promoting transcription factors.

A variety of studies have examined *MDM2* amplification and over-expression in soft tissue tumours.[14–17] These studies, together with studies on other tumour types, have demonstrated that MDM2 over-expression as assessed by immunohistochemical staining is frequently found in the absence of *MDM2* gene amplification,[14,18,19] and that MDM2 over-expression may also be absent in tumours in which amplification has been demonstrated by Southern analysis.[14] These latter observations raise the possibility that gene amplification on chromosome 12q13 may target a gene or genes other than *MDM2* for over-expression. We therefore sought to further investigate the role of the *MDM2* gene in soft tissue tumour development by analyzing a large series of malignant and benign tumours for *MDM2* amplification using Southern analysis and scanning densitometry, and MDM2 and p53 over-expression was examined in a subgroup of these tumours using immunohistochemical staining of frozen sections.

### Materials and methods

### *Clinical samples and cell lines*

Fresh specimens of primary soft tissue tumours were obtained during surgical resection from the Royal Marsden Hospital, London and Surrey, St Thomas's Hospital, London and the Royal Orthopaedic Hospital, Birmingham. Samples were immediately snap frozen in liquid nitrogen and stored at $-70^{\circ}$C until processed. Human cell lines with the exception of RMS[20] were obtained from the American Type Culture Collection (ATCC) and maintained as recommended by their supplier.

### *Southern analysis*

DNA was extracted using a modification of a method described by Steffen and Weinberg.[21] Genomic DNA (10 $\mu$g) was digested with a three- to five-fold excess of HindIII or *Eco*RI, subjected to electrophoresis in 0.8% agarose gels and transferred to Hybond-N filters (Amersham) according to the manufacturer's instructions. Filters were hybridized according to published protocols[22] to $\alpha$-$^{32}$P-labelled probes prepared using random oligonucleotide primers.[23] Filters were washed at 65°C to a final stringency of 0.1 × SSC, 0.1% SDS.

Amplification of the *MDM2* gene was assessed using a 1.5-kb *MDM2* probe prepared by PCR amplification of reverse-transcribed normal fibroblast RNA using the following primers: GGGAGCTCCTCGCCACCATGGTGAGGAG-CAGGCAAATG and GGGGTACCCTCATAG-ACAGGTCAACTAGGGG. The PCR product was subcloned into the pBluescript vector and characterized by sequencing of its entire length. Following hybridization to the *MDM2* probe, blots were stripped by immersion in 0.1% SDS at 95°C and reprobed with p*DCC*1.0 as a control to correct for differences between tumour samples in DNA loading and Southern transfer.[4] The *DCC* gene probe used was chosen as a control because allele loss at the *DCC* locus is found in only around 10% of soft tissue sarcomas, and homozygous deletion has not been demonstrated.[24] Allele loss without reduplication at the *DCC* locus in tumour DNA would result in a two-fold overestimation of the degree of *MDM2* amplification, and this error will be introduced in fewer than 10% of the tumours examined. The degree of amplification was quantified with a Joyce-Loebel Chromoscan 3 scanning densitometer, using absorbance at 530 nm. To allow for inaccuracies due to the effects of stromal contamination, and for the non-linear response of the radiographic film, only samples showing greater than five-fold amplification were considered to be amplified.

### *Immunohistochemical staining of tumour specimens for MDM2 and p53 expression*

Two anti-MDM2 mouse monoclonal antibodies MDM2 (clone IF2) (David Hill, Oncogene Science Inc.) and SMP14,[25] and the p53 antibody DO1 (Oncogene Science Inc.) were used to stain frozen sections of primary tumour specimens using a standard peroxidase-conjugated streptavidin-biotin method (ABC). Slides omitting the first antibody were used as negative controls in all cases. Two tumours known to be positive for either MDM2 or p53 were included as positive controls in each batch of staining. The degree of immunostaining was graded as follows: (negative) $<20\%$ of cells stained positively, (+) 20–50% of cells stained positively, (+ +) $>50\%$ of cells stain positively.

### Results

One hundred and twenty-nine tumours (124 primary tumours and five cell lines) including 29 leiomyosarcomas, 17 liposarcomas, 16 MFHs, 16 rhabdomyosarcomas, 12 malignant peripheral nerve sheath tumours (MPNSTs), 10 synovial sarcomas, 3 osteosarcomas, 2 fibrosarcomas, 2 chondrosarcomas, 7 other sarcomas, 5 fibromatoses and 10 benign soft tissue neoplasms were analyzed. Greater than five-fold amplification of *MDM2* was detected in 14/114 (12.3%) of the malignant tumours but in none of the fibromatoses or benign tumours. The positive samples included 3/16 (19%) MFHs (STS11, STS41 and STS140), 4/17 (24%) liposarcomas (STS20, STS44, STS61 and STS131), 2/29 (7%) leiomyosarcomas (STS87 and STS320), 2/16 (12.5%) rhabdomyosarcomas (1 embryonal rhabdomyosarcoma STS93 and the alveolar rhabdomyosarcoma cell line RMS), 2/12 (17%) MPNSTs (STS52 and STS102) and a single

**Table 1.** *Amplification of the* MDM2 *gene in soft tissue tumours*

| Tumour type | Number examined | Number. amplified | Percentage amplified |
|---|---|---|---|
| Leiomyosarcoma | 29 | 2 | 7 |
| MFH | 16 | 3 | 19 |
| Liposarcoma | 17 | 4 | 24 |
| Rhabdomyosarcoma | 16 | 2 | 12.5 |
| MPNST | 12 | 2 | 17 |
| Synovial sarcoma | 10 | 0 | 0 |
| Other sarcomas* | 14 | 1 | 7 |
| Fibromatosis | 5 | 0 | 0 |
| Benign tumours** | 10 | 0 | 0 |

*Includes 3 osteosarcomas, 2 chondrosarcomas, 2 fibrosarcomas (one of which showed *MDM2* amplification), 1 sarcoma NOS, 2 clear cell sarcomas, 1 dermatofibrosarcoma proturberans, 1 post-irradiation spindle cell sarcoma, 1 neuroepithelioma and 1 renal leiomyoblastoma.

**Includes 3 haemangiomas, 3 neurofibromas, 2 neurilemmomas, 1 paraganglioma and 1 angiolipoma.

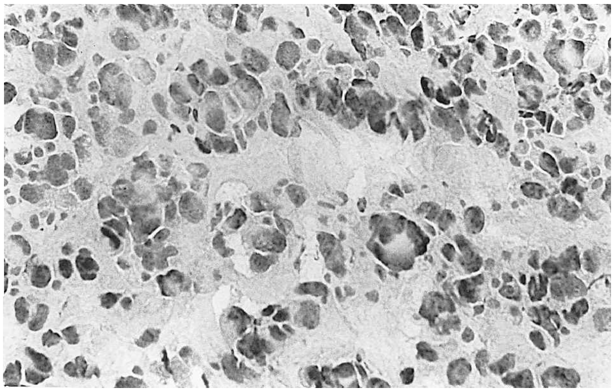

**Fig. 2.** *Immunohistochemical staining of primary tumours for MDM2 over-expression. Immunohistochemical staining of STS24, a high-grade MFH, using the mouse monoclonal MDM2 antibody MDM2 (IF2) (Oncogene Science). Greater than 50% of the cells show positive staining for MDM2.*

fibrosarcoma (STS49). These results are presented in Table 1, and examples of *MDM2* gene amplification are illustrated in Fig. 1. STS162, a recurrence of the liposarcoma STS44, was not included in the original group of tumours but was subsequently analyzed, and also demonstrated *MDM2* gene amplification.

Thirty-nine of the tumours analyzed for *MDM2* amplification were also examined for MDM2 over-expression by immunohistochemical staining (Table 2). These tumours were examined without knowledge of the *MDM2* gene amplification results, but had been selected to include 11 of the tumours with *MDM2* amplification. This group included 8 liposarcomas, 8 MFHs, 6 leiomyosarcomas, 5 MPNSTs, 3 rhabdomyosarcomas, 3 synovial sarcomas, 2 sarcomas NOS (not otherwise specified), 1 clear cell sarcoma, 1 fibrosarcoma and 2 fibromatoses. Of these, 21/39 (54%) including 5 MFHs, 4 liposarcomas (including STS44 and its recurrence STS162), 3 MPNSTs, 3 rhabdomyosarcomas, 2 leiomyosarcomas, 2 synovial sarcomas, 1 fibrosarcoma and 1 clear cell sarcoma showed positive immunohistochemical staining for MDM2. An example is shown in Fig. 2. All 11 of the tumours in which the *MDM2* gene was amplified stained positively for MDM2 over-expression, and MDM2 over-expression was confirmed in the RMS cell line by Northern analysis. Conversely, the *MDM2* gene was amplified in 0/18 (0%) of the negatively staining tumours. Of the six tumours with strongly positive staining for MDM2 (>50% of cells positive), 5/6 (83%) demonstrated *MDM2* amplification, whereas only 6/15 (40%) of the relatively weakly staining tumours (20–50% cells positive) demonstrated *MDM2* amplification. The level of amplification in weakly staining tumours was, however, comparable to that seen in the more strongly staining tumours. Among five additional tumours, for which data regarding *MDM2* gene amplification were not available, over-expression was identified in two MFHs, STS7 and STS9.

p53 over-expression, as assessed by immunohistochemical staining, was identified in four tumours. In two of these tumours, STS24 and STS102, the *MDM2* gene was also over-expressed, and in one of these tumours, STS102, a malignant peripheral nerve sheath tumour, *MDM2* gene amplification was found.

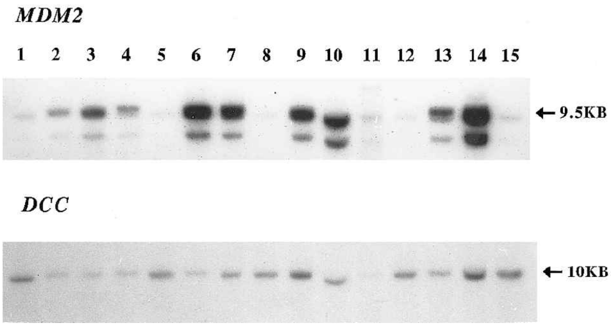

**Fig. 1.** *Amplification of the* MDM2 *gene in malignant soft tissue tumours. Southern blot analysis of* Eco*RI-digested DNA probed sequentially with p*DCC*1.0 and an* MDM2 *cDNA probe, demonstrating* MDM2 *gene amplification in several tumours. The degree of gene amplification was estimated by using scanning densitometric analysis of the resulting autoradiographs. The loading is as follows: lane 1, normal leucocyte DNA; lane 2, RMS cell line; lane 3, STS93; lane 4, STS102; lane 5, normal leucocyte DNA; lane 6, STS49; lane 7, STS11; lane 8, normal leucocyte DNA; lane 9, STS41; lane 10, STS44; lane 11, STS61; lane 12, normal leucocyte DNA; lane 13, STS140; lane 14, STS162; lane 15, normal leucocyte DNA.*

**Table 2.** *Amplification and/or over-expression of the* MDM2 *gene in human soft tissue sarcomas*

| Tumour | Histology | Grade | Source | *MDM2* gene amplification* | MDM2 over-expression** |
|---|---|---|---|---|---|
| STS7 | MFH | High | Recurrence | NE | + |
| STS9 | MFH | High | Recurrence | NE | + |
| STS11 | MFH | High | Recurrence | 36 | + |
| STS20 | Liposarcoma | Low | Primary | 7 | NE |
| STS23 | Pleomorphic rhabdomyosarcoma | High | Primary | ND | + |
| STS24 | MFH | High | Recurrence | ND | + + |
| STS32 | Clear cell sarcoma | High | Primary | ND | + |
| STS34 | Leiomyosarcoma | High | Primary | ND | + |
| STS41 | MFH | Low | Recurrence | 14 | + |
| STS44 | Liposarcoma | High | Recurrence | 33 | + |
| STS49 | Fibrosarcoma | Low | Recurrence | > 50 | + + |
| STS52 | MPNST | High | Primary | 6 | + |
| STS56 | MFH | High | Recurrence | ND | + |
| STS61 | Liposarcoma | Intermediate | Recurrence | 44*** | + |
| STS62 | MPNST | High | Primary | ND | + |
| STS69 | Alveolar rhabdomyosarcoma | High | Primary | ND | + |
| STS87 | Leiomyosarcoma | Low | Metastasis | > 50 | + + |
| STS93 | Embryonal rhabdomyosarcoma | High | Metastasis | 50 | + + |
| STS102 | MPNST | Low | Recurrence | 20 | + + |
| STS114 | Synovial sarcoma | High | Primary | ND | + |
| STS117 | Liposarcoma | High | Recurrence | ND | + |
| STS125 | Synovial sarcoma | High | Primary | ND | + |
| STS131 | Liposarcoma | Low | Primary | 48 | NE |
| STS140 | MFH | High | Primary | 34 | + |
| STS162 | Liposarcoma | High | Recurrence | 28 | + + |
| STS320 | Leiomyosarcoma | High | Primary | > 50 | NE |
| RMS | Rhabdomyosarcoma | — | Cell line | 14 | + [+] |

*Amplification was detected by Southern blot analysis using an *MDM2* cDNA probe spanning the whole open reading frame. Scanning densitometry was used to compare the signal to that obtained with the probe p*DCC1*.0.

**Detected by immunohistochemical staining of frozen sections with the monoclonal antibody MDM2 (Ab-1) (Oncogene Science). Staining was graded (+) 20–50% cells stain positively, (+ +) > 50% of cells stain positively.

***Problems with DNA degradation and digestion with this sample impair the accuracy of this result.

[+] Northern analysis.

NE = not evaluated; ND = not detected.

### Discussion

This study confirms the observation of amplification of the *MDM2* gene in soft tissue sarcomas which was first presented by Oliner *et al.*,[4] and extends the original observation of *MDM2* amplification in MFHs, liposarcomas and osteosarcomas, to include leiomyosarcomas, MPNSTs, a primary embryonal rhabdomyosarcoma and an alveolar rhabdomyosarcoma cell line (RMS). In contrast to Oliner *et al.*[4] who detected *MDM2* amplification in 36% (17/47) of their malignant sarcomas, we have detected amplification in only 12.3% of this sample of malignant tumours. These results are, however, in keeping with the results of Cordon-Cardo *et al.*[14] who detected a greater than five-fold amplification of the *MDM2* gene in only 15% (11/73) of their sarcomas, with Forus *et al.*[7] who detected *MDM2* amplification in 9% (9/98) of their sarcomas, and with the results of Ladanyi *et al.*[26] who detected *MDM2* amplification in 14% (4/28) of their high-grade osteosarcomas.

*MDM2* amplification was seen in both high- and low-grade tumours, as well as in primary, recurrent and metastatic tumours (Table 2). There did not appear to be any clear correlation between the degree of amplification and the histological subtype or grade, or whether the source of the tumour specimen was a primary, recurrent or metastatic tumour. These results indicate that amplification of the *MDM2* gene may be important in the development of a significant minority of a wide variety of histological subtypes of malignant soft tissue tumour, but they provide no support for a role for *MDM2* gene amplification in the development of benign soft tissue neoplasms, or locally aggressive tumours such as fibromatoses.

Whereas only 11/39 of the tumours selected for MDM2 immunohistochemical staining possessed *MDM2* gene amplification, 21/39 of the tumours in this subgroup analyzed were immunostain positive for MDM2 over-expression. Other studies are in broad agreement with these results. Cordon-Cardo

*et al.*[14] identified MDM2 over-expression in 37% (76/207) of their sarcomas, with gene amplification present in only 15% (11/73) of a subset of these tumours, and Lianes *et al.*[18] demonstrated MDM2 over-expression in 26/87 human bladder tumours, but only 1/26 of these tumours demonstrated *MDM2* gene amplification. These results suggest that mechanisms other than gene amplification may be important in raising cellular levels of the MDM2 protein; for example, alterations in the stability of the MDM2 protein, or in the rate of transcription or translation or of the stability of the MDM2 mRNA.

Only 6/11 of the tumours with *MDM2* gene amplification reported by Cordon-Cardo *et al.*[14] showed positive immunohistochemical staining (>20% cells positive) for MDM2 over-expression, raising the possibility that in some sarcomas the target for gene amplification on chromosome 12q13 may not be *MDM2*. The results presented here, however, have demonstrated that *MDM2* amplification accurately predicts for MDM2 over-expression. This complete correlation is corroborated by three other studies with soft tissue tumours. Leach *et al.*[15] identified *MDM2* gene amplification and MDM2 over-expression in 8/24 MFHs and liposarcomas, Florenes *et al.*[16] demonstrated *MDM2* gene amplification and over-expression in 10/98 bone and soft tissue tumours, and Nakayama *et al.*[17] identified *MDM2* gene amplification, and over-expression by Northern analysis, in each of 11/48 soft tissue tumours. These observations, the demonstration that *MDM2* inhibits transactivation by the product of the p53 gene (a gene known to contribute to the neoplastic process in sarcomas) and the demonstration that *MDM2* is a dominantly transforming oncogene[9] provide strong evidence in favour of a role for *MDM2* amplification and over-expression in the genesis of a subgroup of soft tissue sarcomas.

We have identified over-expression of both p53 and MDM2 in two tumours, STS24 and STS102, one of which possessed *MDM2* gene amplification. The status of the p53 gene in these two tumours has not been examined. However, when similar findings have been demonstrated in other tumours,[16,18] over-expression of the MDM2 protein was accompanied by over-expression of wild-type and not mutant p53 protein. For example, Landers *et al.*[27] examined choriocarcinoma cell lines and found over-expression of both wild-type p53 and MDM2. The over-expression of MDM2 was not explained by gene amplification, elevated transcription or altered protein stability, but appeared to have resulted from increased protein translation. In addition to these observations, the level of *MDM2* transcription is known to be regulated by the expression of wild-type p53.[11] Additional data regarding the status of the p53 gene were available for 38 of the tumours examined for *MDM2* gene amplification (29 leiomyosarcomas, 4 cell lines, 1 primary rhabdomyosarcoma, 2 MFHs, 1 liposarcoma and 1 post-irradiation spindle cell tumour), and nine of these had also been examined for MDM2 over-expression. Thirteen tumours possessed p53 mutations, seven had undergone homozygous rearrangement of the p53 gene and six had mis-sense point mutations.[24,28] Two of these tumours, STS7, an MFH, and STS117, a liposarcoma, both of which had undergone homozygous rearrangement at the p53 locus, also demonstrated MDM2 over-expression. In STS117 the over-expression was found in the absence of gene amplification. The status of wild-type p53 protein expression in tumour cells may, therefore, modulate the level of MDM2 protein expression, thus providing a mechanism for MDM2 over-expression in the absence of gene amplification in a subgroup of tumour cells.

### Acknowledgements

We would like to thank Professor Bert Vogelstein for providing the probe p*DCC*1.0, and Dr Alasdair Stamps for providing the *MDM2* cDNA probe. The mouse monoclonal antibody MDM2 (IF2) was a kind gift from David Hill, Oncogene Science Inc. This study was supported by grants from the Cancer Research Campaign and the Medical Research Council.

### References

1 Mandahl N, Heim S, Willen H, *et al.* Characteristic karyotypic anomalies identify subtypes of malignant fibrous histiocytoma. *Genes Chrom Cancer* 1989; 1:9–14.
2 Mertens F, Johansson B, Mandahl N, *et al.* Clonal chromosome abnormalities in two liposarcomas. *Cancer Genet Cytogenet* 1987; 28:137–44.
3 Wang-Wuu S, Soukop S, Ballard E, *et al.* Chromosomal analysis of sixteen human rhabdomyosarcomas. *Cancer Res* 1988; 48:983–7.
4 Oliner JD, Kinzler KW, Meltzer PS, *et al.* Amplification of a gene encoding a p53-associated protein in human sarcomas. *Nature* 1992; 358:80–3.
5 Kinzler KW, Ruppert JM, Bigner SH, *et al.* The GLI gene is a member of the *Kruppel* family of zinc finger proteins. *Nature* 1988; 332:371–4.
6 Khatib ZA, Matsushime H, Valentine M, *et al.* Coamplification of the *CDK4* gene with the *MDM2* and *GLI* in human sarcomas. *Cancer Res* 1993; 53:5535–41.
7 Forus A, Florenes VA, Maelandsmo GM, *et al.* Mapping of amplification units in the q13–14 region of chromosome 12 in human sarcomas: some amplica do not include *MDM2*. *Cell Growth Diff* 1993; 4:1065–70.
8 Jankowski SA, Mitchell DS, Smith SH, *et al.* *SAS*, a gene amplified in human sarcomas, encodes a new member of the transmembrane 4 superfamily of proteins. *Oncogene* 1994; 9:1205–11.
9 Fakharzedeh SS, Trusko SP, George DL. Tumourigenic potential associated with enhanced expression of a gene that is amplified in a mouse tumour cell line. *EMBO J* 1991; 10:1565–9.
10 Momand J, Zambetti GP, Olsen DC, *et al.* The MDM2 oncogene product forms a complex with the p53 protein and inhibits p53-mediated transactivation. *Cell* 1992; 69:1237–45.

11 Wu X, Bayle JH, Olsen D, *et al.* The p53–MDM2 auto-regulatory feedback loop. *Genes Dev* 1993; 7:1126–32.

12 Xiao Z-X, Chen J, Levine AJ, *et al.* Interaction between the retinoblastoma protein and the oncoprotein MDM2. *Nature* 1995; 375:694–8.

13 Martin K, Trouche D, Hagemeier C, *et al.* Stimulation of E2F1/DP1 transcriptional activity by MDM2 oncoprotein. *Nature* 1995; 375:691–4.

14 Cordon-Cardo C, Latres E, Drobnjak M, *et al.* Molecular abnormalities of *MDM2* and p53 genes in adult soft tissue sarcomas. *Cancer Res* 1994; 54:794–9.

15 Leach FS, Tokino T, Meltzer P, *et al.* p53 mutation and *MDM2* amplification in human soft tissue sarcomas. *Cancer Res* 1993; 53:2231–4.

16 Florenes VA, Maelandsmo GM, Forus A, *et al.* *MDM2* gene amplification and transcript levels in human sarcomas: relationship to p53 gene status. *J Natl Cancer Inst* 1994; 86:1297–1302.

17 Nakayama T, Toguchida, Wadayama BI, *et al.* *MDM2* gene amplification in bone and soft tissue tumours: association with tumour progression in differentiated adipose-tissue tumours. *Int J Cancer* 1995; 64:342–6.

18 Lianes P, Orlow I, Zhang ZF, *et al.* Altered patterns of MDM2 and p53 expression in human blader cancer. *J Natl Cancer Inst* 1994; 86:1325–30.

19 Bueso Ramos CE, Yang Y, deLeon E, *et al.* The human MDM2 gene is over-expressed in leukaemias. *Blood* 1993; 82:2617–23.

20 Garvin AJ, Stanley WS, Bennett DD, *et al.* The *in vitro* growth, heterotransplantation, and differentiation of a human rhabdomyosarcoma cell line. *Am J Pathol* 1986; 125:208–17.

21 Steffen D, Weinberg RA. The integrated genome of murine leukaemia virus. *Cell* 1978; 15:1003–10.

22 Church GM, Gilbert W. Genomic sequencing. *Proc Natl Acad Sci* 1984; 81:1991–5.

23 Feinberg AP, Vogelstein B. A technique for radiolabelling DNA restriction endonuclease fragments to high specific activity. *Anal Biochem* 1983; 132:6–13.

24 Patterson H, Gill S, Fisher C, *et al.* Abnormalities of the p53, *MDM2* and *DCC* genes in human leiomyosarcomas. *Br J Cancer* 1994; 69:1052–8.

25 Pickersley SM, Vojtesek B, Sparks A, *et al.* Immunohistochemical analysis of the interaction of p53 with MDM2; fine mapping of the MDM2 binding site on p53 using synthetic peptides. *Oncogene* 1994; 9:2523–9.

26 Ladanyi M, Cha C, Lewis R, *et al.* *MDM2* gene amplification in metastatic osteosarcoma. *Cancer Res* 1993; 53:16–18.

27 Landers JE, Haines DS, Strauss JF III, *et al.* Enhanced translation: a novel mechanism of *MDM2* oncogene over-expression identified in human tumour cells. *Oncogene* 1994; 9:2745–50.

28 Stratton MR, Moss S, Warren W, *et al.* Mutation of the p53 gene in human soft tissue sarcomas: association with abnormalities of the RB1 gene. *Oncogene* 1990; 5:1297–301.

## References

[PDF_00553] Bueso-Ramos C. E., Yang Y., deLeon E., McCown P., Stass S. A., Albitar M. (1993). The human MDM-2 oncogene is overexpressed in leukemias.. Blood.

[PDF_00562] Church G. M., Gilbert W. (1984). Genomic sequencing.. Proc Natl Acad Sci U S A.

[PDF_00535] Cordon-Cardo C., Latres E., Drobnjak M., Oliva M. R., Pollack D., Woodruff J. M., Marechal V., Chen J., Brennan M. F., Levine A. J. (1994). Molecular abnormalities of mdm2 and p53 genes in adult soft tissue sarcomas.. Cancer Res.

[PDF_00517] Fakharzadeh S. S., Trusko S. P., George D. L. (1991). Tumorigenic potential associated with enhanced expression of a gene that is amplified in a mouse tumor cell line.. EMBO J.

[PDF_00564] Feinberg A. P., Vogelstein B. (1983). A technique for radiolabeling DNA restriction endonuclease fragments to high specific activity.. Anal Biochem.

[PDF_00541] Flørenes V. A., Maelandsmo G. M., Forus A., Andreassen A., Myklebost O., Fodstad O. (1994). MDM2 gene amplification and transcript levels in human sarcomas: relationship to TP53 gene status.. J Natl Cancer Inst.

[PDF_00508] Forus A., Flørenes V. A., Maelandsmo G. M., Meltzer P. S., Fodstad O., Myklebost O. (1993). Mapping of amplification units in the q13-14 region of chromosome 12 in human sarcomas: some amplica do not include MDM2.. Cell Growth Differ.

[PDF_00556] Garvin A. J., Stanley W. S., Bennett D. D., Sullivan J. L., Sens D. A. (1986). The in vitro growth, heterotransplantation, and differentiation of a human rhabdomyosarcoma cell line.. Am J Pathol.

[PDF_00513] Jankowski S. A., Mitchell D. S., Smith S. H., Trent J. M., Meltzer P. S. (1994). SAS, a gene amplified in human sarcomas, encodes a new member of the transmembrane 4 superfamily of proteins.. Oncogene.

[PDF_00505] Khatib Z. A., Matsushime H., Valentine M., Shapiro D. N., Sherr C. J., Look A. T. (1993). Coamplification of the CDK4 gene with MDM2 and GLI in human sarcomas.. Cancer Res.

[PDF_00502] Kinzler K. W., Ruppert J. M., Bigner S. H., Vogelstein B. (1988). The GLI gene is a member of the Kruppel family of zinc finger proteins.. Nature.

[PDF_00575] Ladanyi M., Cha C., Lewis R., Jhanwar S. C., Huvos A. G., Healey J. H. (1993). MDM2 gene amplification in metastatic osteosarcoma.. Cancer Res.

[PDF_00578] Landers J. E., Haines D. S., Strauss J. F., George D. L. (1994). Enhanced translation: a novel mechanism of mdm2 oncogene overexpression identified in human tumor cells.. Oncogene.

[PDF_00538] Leach F. S., Tokino T., Meltzer P., Burrell M., Oliner J. D., Smith S., Hill D. E., Sidransky D., Kinzler K. W., Vogelstein B. (1993). p53 Mutation and MDM2 amplification in human soft tissue sarcomas.. Cancer Res.

[PDF_00550] Lianes P., Orlow I., Zhang Z. F., Oliva M. R., Sarkis A. S., Reuter V. E., Cordon-Cardo C. (1994). Altered patterns of MDM2 and TP53 expression in human bladder cancer.. J Natl Cancer Inst.

[PDF_00489] Mandahl N., Heim S., Willén H., Rydholm A., Eneroth M., Nilbert M., Kreicbergs A., Mitelman F. (1989). Characteristic karyotypic anomalies identify subtypes of malignant fibrous histiocytoma.. Genes Chromosomes Cancer.

[PDF_00532] Martin K., Trouche D., Hagemeier C., Sørensen T. S., La Thangue N. B., Kouzarides T. (1995). Stimulation of E2F1/DP1 transcriptional activity by MDM2 oncoprotein.. Nature.

[PDF_00493] Mertens F., Johansson B., Mandahl N., Heim S., Bennet K., Rydholm A., Willén H., Mitelman F. (1987). Clonal chromosome abnormalities in two liposarcomas.. Cancer Genet Cytogenet.

[PDF_00521] Momand J., Zambetti G. P., Olson D. C., George D., Levine A. J. (1992). The mdm-2 oncogene product forms a complex with the p53 protein and inhibits p53-mediated transactivation.. Cell.

[PDF_00545] Nakayama T., Toguchida J., Wadayama B., Kanoe H., Kotoura Y., Sasaki M. S. (1995). MDM2 gene amplification in bone and soft-tissue tumors: association with tumor progression in differentiated adipose-tissue tumors.. Int J Cancer.

[PDF_00499] Oliner J. D., Kinzler K. W., Meltzer P. S., George D. L., Vogelstein B. (1992). Amplification of a gene encoding a p53-associated protein in human sarcomas.. Nature.

[PDF_00567] Patterson H., Gill S., Fisher C., Law M. G., Jayatilake H., Fletcher C. D., Thomas M., Grimer R., Gusterson B. A., Cooper C. S. (1994). Abnormalities of the p53 MDM2 and DCC genes in human leiomyosarcomas.. Br J Cancer.

[PDF_00570] Picksley S. M., Vojtesek B., Sparks A., Lane D. P. (1994). Immunochemical analysis of the interaction of p53 with MDM2;--fine mapping of the MDM2 binding site on p53 using synthetic peptides.. Oncogene.

[PDF_00560] Steffen D., Weinberg R. A. (1978). The integrated genome of murine leukemia virus.. Cell.

[PDF_00582] Stratton M. R., Moss S., Warren W., Patterson H., Clark J., Fisher C., Fletcher C. D., Ball A., Thomas M., Gusterson B. A. (1990). Mutation of the p53 gene in human soft tissue sarcomas: association with abnormalities of the RB1 gene.. Oncogene.

[PDF_00496] Wang-Wuu S., Soukup S., Ballard E., Gotwals B., Lampkin B. (1988). Chromosomal analysis of sixteen human rhabdomyosarcomas.. Cancer Res.

[PDF_00526] Wu X., Bayle J. H., Olson D., Levine A. J. (1993). The p53-mdm-2 autoregulatory feedback loop.. Genes Dev.

[PDF_00529] Xiao Z. X., Chen J., Levine A. J., Modjtahedi N., Xing J., Sellers W. R., Livingston D. M. (1995). Interaction between the retinoblastoma protein and the oncoprotein MDM2.. Nature.

